# Supplementary material for: Modulating hierarchical self-assembly in thermoresponsive intrinsically disordered proteins through high-temperature incubation time
Source: Sci Rep. 2023 Dec 7;13:21688. doi: 10.1038/s41598-023-48483-w (PMC10709347; doi:10.1038/s41598-023-48483-w)
Supplement: Supplementary file 1 — Supplementary Information. [file 41598_2023_48483_MOESM1_ESM.pdf]

Modulating Hierarchical Self-Assembly in Thermoresponsive Intrinsically Disordered Proteins Through High-Temperature Incubation Time

Vaishali Sethi[1,2,3], *Dana Cohen-Gerassi*[2,3,4], *Sagi Meir*[1,2,3], *Max Ney*[5], *Yulia Shmidov*[5],*Gil Koren*[1,23,], *Lihi Adler-Abramovich*[2,3,4], *Ashutosh Chilkoti*[5], *and Roy Beck*[*1,2,3]

[1] School of Physics and Astronomy, Tel Aviv University, Tel Aviv 6997801, Israel.

[2] The Center for Physics and Chemistry of Living Systems, Tel Aviv University, Tel Aviv 6997801, Israel.

[3] The Center for Nanoscience and Nanotechnology, Tel Aviv University, Tel Aviv 6997801, Israel.

[4] Department of Oral Biology, The Goldschleger School of Dental Medicine, Sackler Faculty of Medicine, Tel Aviv University, Tel Aviv 6997801, Israel.

[5] Department of Biomedical Engineering, Duke University, Durham, NC 27708, USA

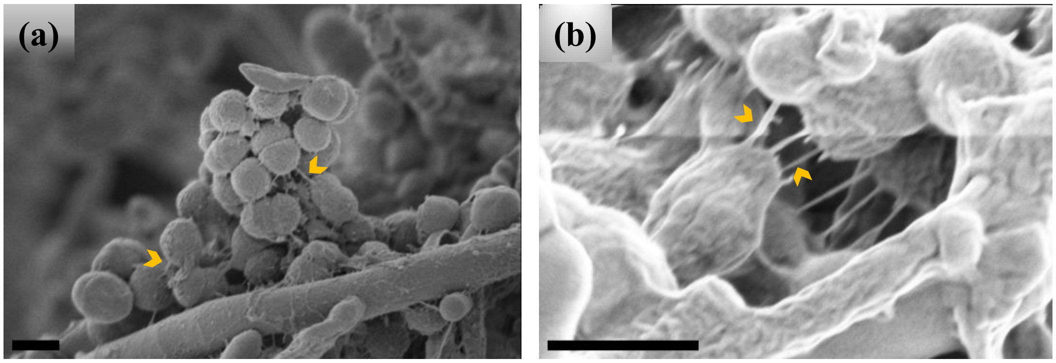

Figure S1. HRSEM images of (a) The $[WT]_{40}$-48 h and (b) $[WT]_{80}$-24 h system exhibiting interconnecting nano-fibers (shown by arrowheads), which leads to an alignment of the nano ellipsoids to rods. Scale bar: 500nm.

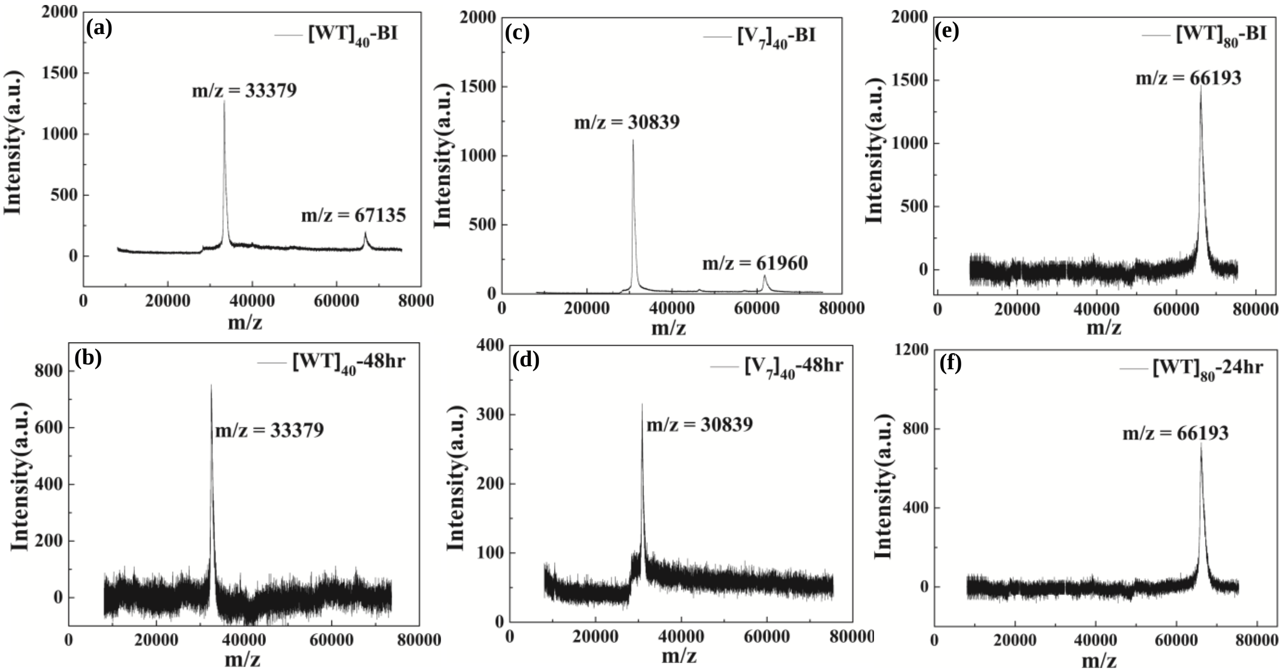

Figure S2. Mass spectrum of different samples before incubation (BI) and after a desired period of incubation (a) $[WT]_{40}$-BI (b) $[WT]_{40}$-48hr (c) $[V_7]_{40}$ -BI (d) $[V_7]_{40}$ -48hr (e) $[WT]_{80}$-BI (f) $[WT]_{80}$-24hr sample.

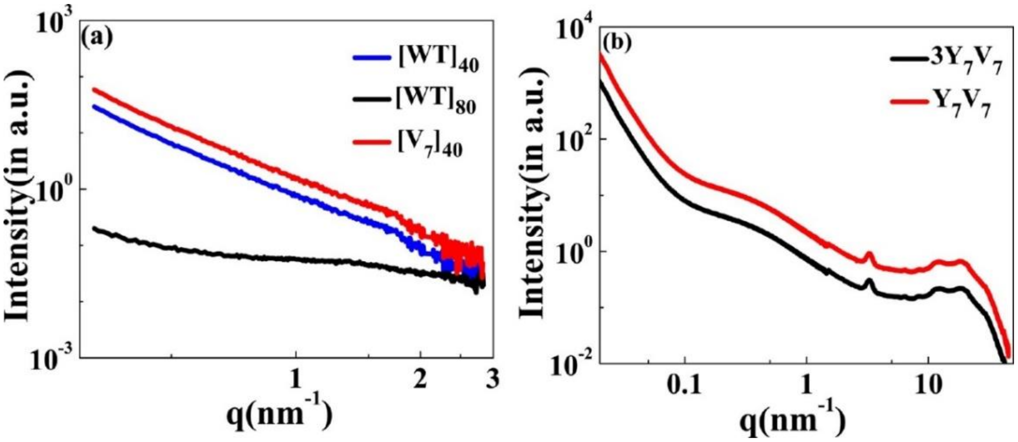

Figure S3. Scattering profiles of (a) $[WT]_{40}$, $[WT]_{80}$, and $[V_7]_{40}$ lyophilized powder. (b) $3Y_7V_7$ and Y_7V_7 systems at t_{80} = 48 h. The lack of any correlation peaks in powders and 25% ($3Y_7V_7$) or 50% (Y_7V_7) V mutated A-IDPs indicates the absence of a short-or-long range order.

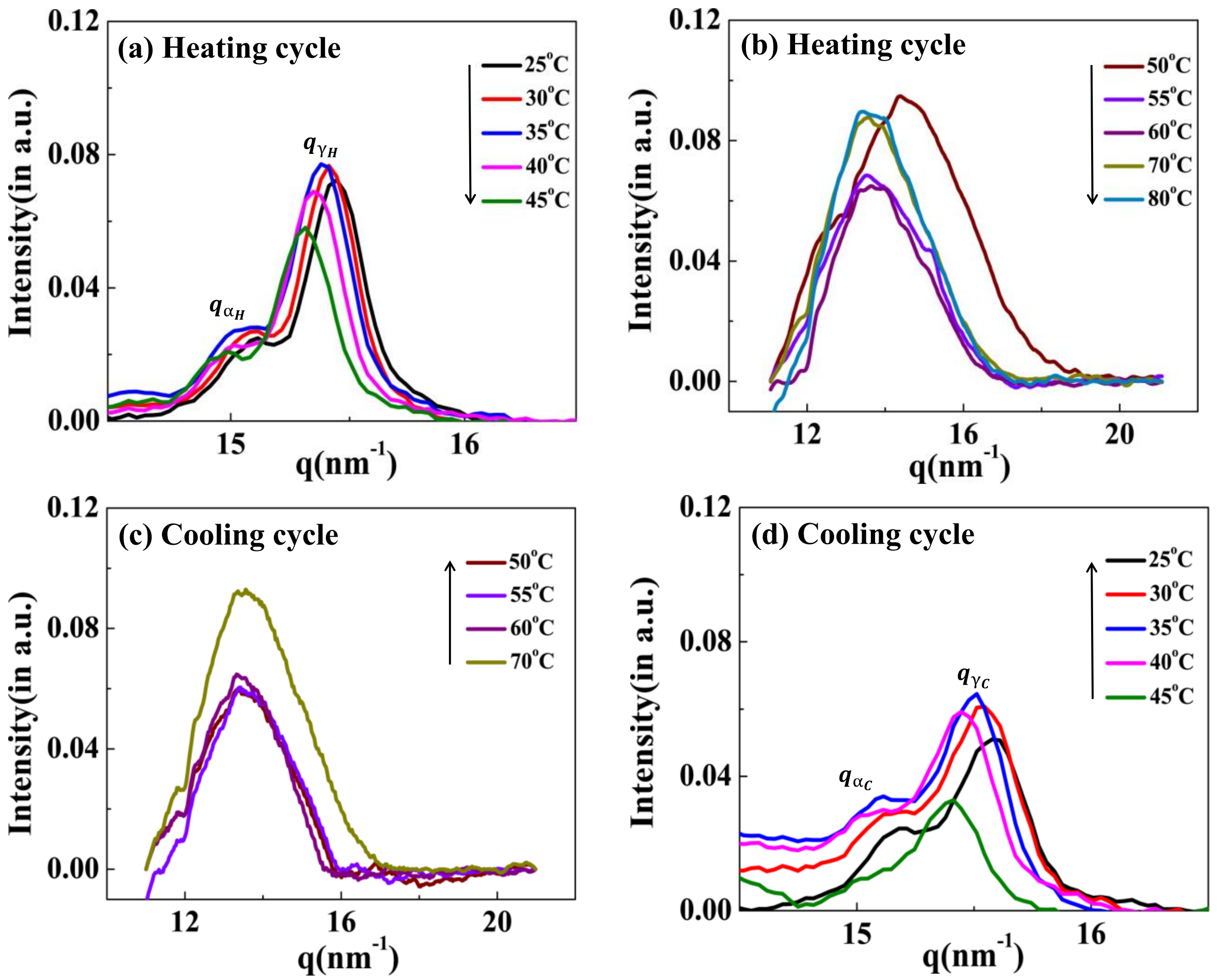

Figure S4. WAXS profiles of $[V_7]_{40}$-48hr sample representing q_α and q_γ correlation peaks during heating cycle (a) from 25°C to 45°C (b) from 50°C to 80°C. During cooling cycle (c) from 70°C to 50°C (d) from 45°C to 25°C. The subscript H and C represents heating and cooling cycle for corresponding q_α and q_γ peaks.

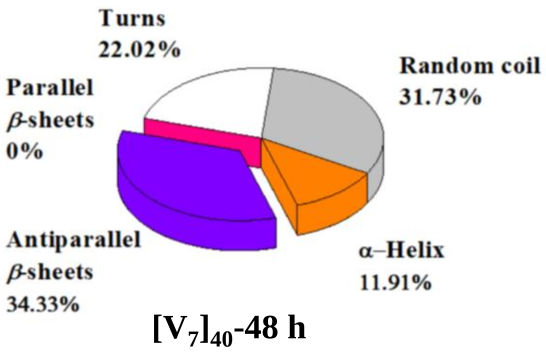

$[V_7]_{40}$-48 h

Figure S5. Pie charts representing the percentage of secondary structure at t_{80} = 48 h in $[V_7]_{40}$ protein sample obtained by quantifying circular dichroism data using BestSel algorithm.[1] The $[V_7]_{40}$ sample exhibits antiparallel β-sheets (~34%), as their major secondary structure in phosphate buffer after 48hrs of incubation.

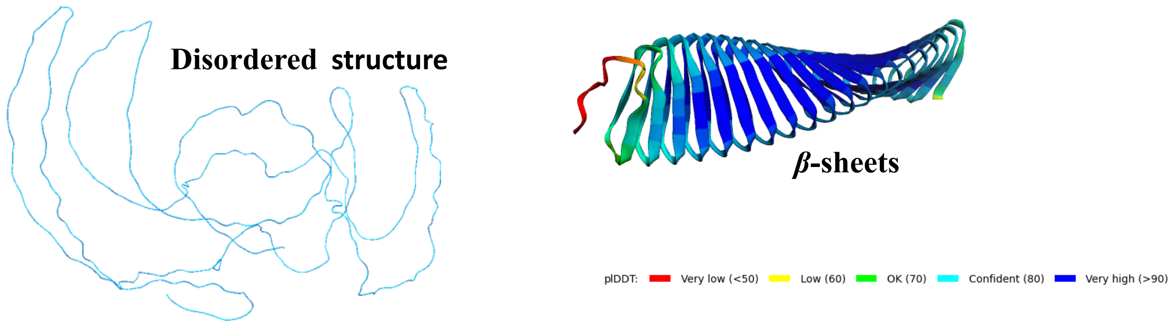

(a) $[WT]_{40}$ (b) $[V_7]_{40}$

Figure S6. The secondary structure predicted by alphafold2[2] (a) for $[WT]_{40}$ system disordered structure (b) for $[V7]_{40}$ system *β*-sheets are respectively predicted.

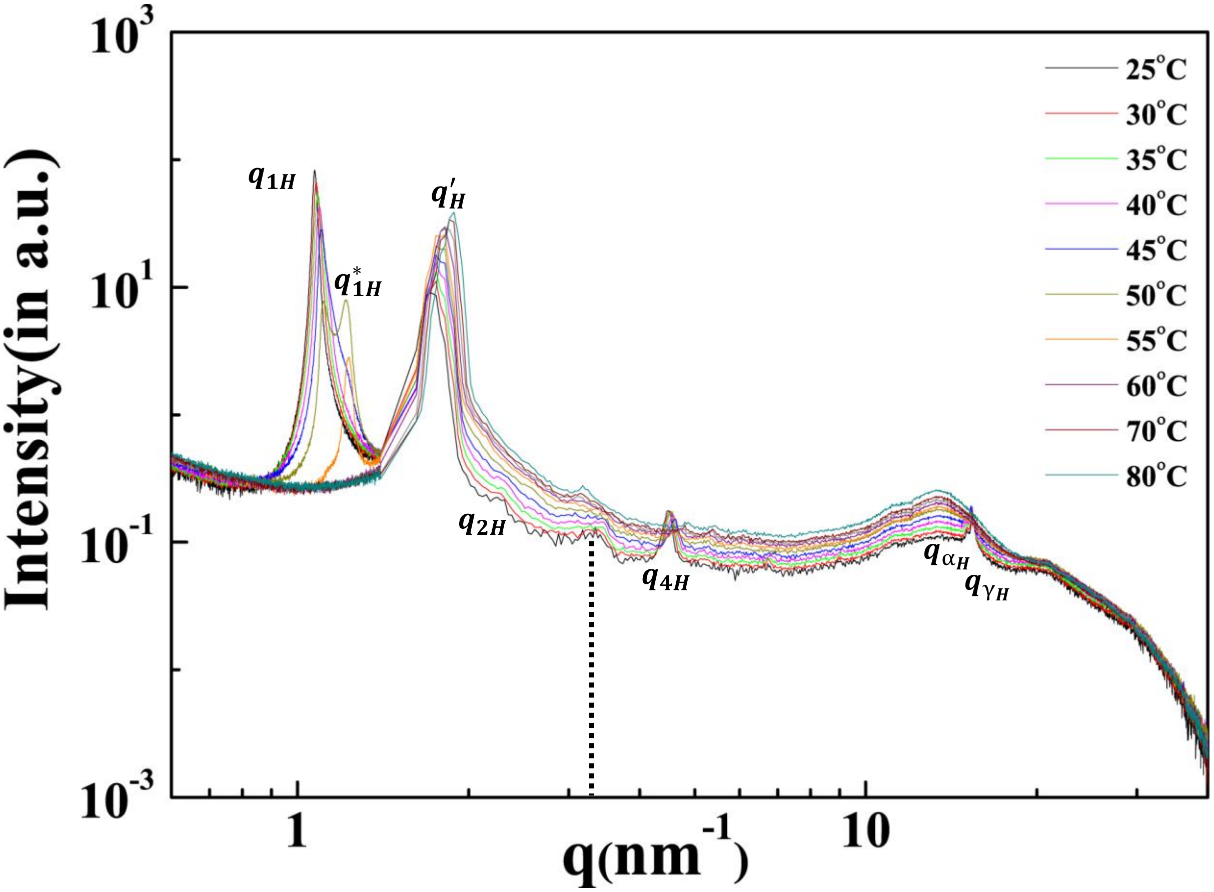

Figure S7. Radially integrated 1D SAXS/WAXS profile of the $[V_7]_{40}$-48 h system during the heating cycle, with changes in correlation peaks as a function of ramping the temperature from 25°C to 80°C with a ramp rate of 5°C. The correlation peaks q_{1_H}, q_{2_H}, and q_{4_H} reflect self-assembled lamellar structures in the $[V_7]_{40}$-48hr system, while the $q_{\alpha H}$ and $q_{\gamma H}$ peaks corresponding to partial side chain ordering. The q_{2_H} and q_{4_H} peaks disappear at 45°C except q_{1_H}, which splits into another peak $q^*_{1_H}$ at 50°C, q_{1_H} and $q^*_{1_H}$ disappear at 50°C and 60°C respectively. The correlation peak q'_H corresponds to short-range order in this system, and it persists at all studied temperatures. The peak at q ~ 3.4 nm^{-1} (represented by a dotted line) corresponds to the residual diffraction peak of the Kapton window of the X-ray system.[3]

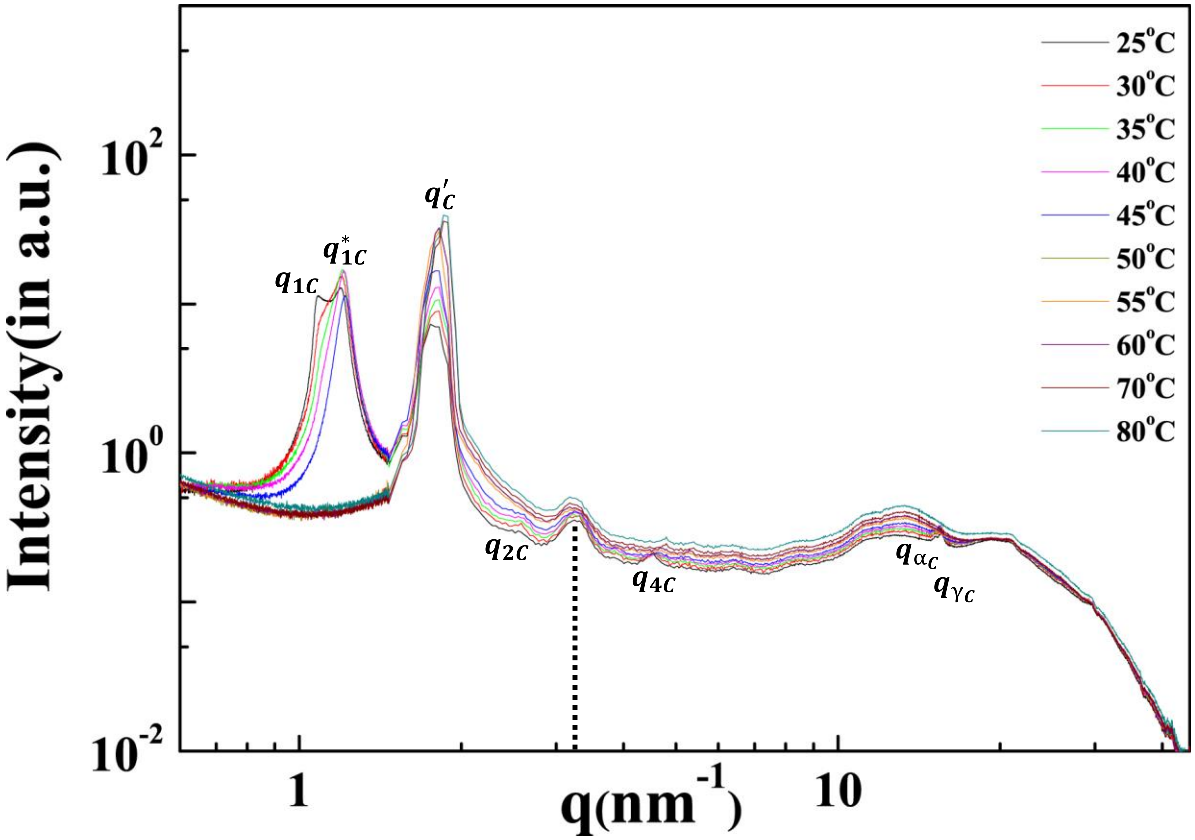

Figure S8. Radially integrated 1D SAXS/WAXS profile of the $[V_7]_{40}$-48 h system during the cooling cycle, exhibiting changes in correlation peaks upon ramping down the temperature from 80°C to 25°C with a ramp rate of 5°C. The correlation peaks $q_{1_C}^*$, q_{2_C} and q_{4_C}, which reappear at 45°C reflect the lamellar structure in the $[V_7]_{40}$-48 hr system, while the $q_{\alpha C}$ *and* $q_{\gamma C}$ peaks corresponding to partial side chain ordering. The q_C' correlation peak reflects the short-range order, different from lamellar nanostructures, and this also persisted throughout the cooling cycle. The peak at q ~3.4 nm^{-1} (shown by a dotted line) corresponds to a residual diffraction peak of the kapton window originating from the experimental setup.[3]

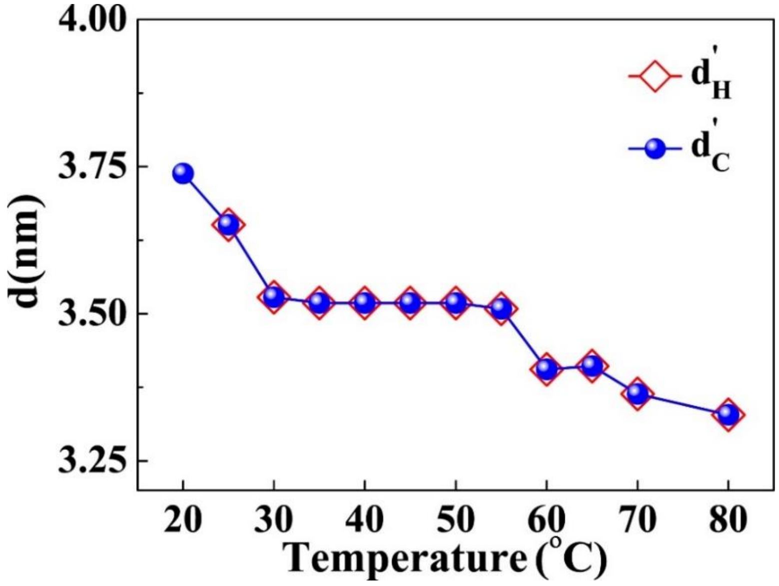

Figure S9. Variation in the interplanar distance (d') corresponding to the correlation peak q' of the $[V_7]_{40}$-48h system as a function of temperature during the heating and cooling cycle. The interplanar distances are independent of cycle direction.

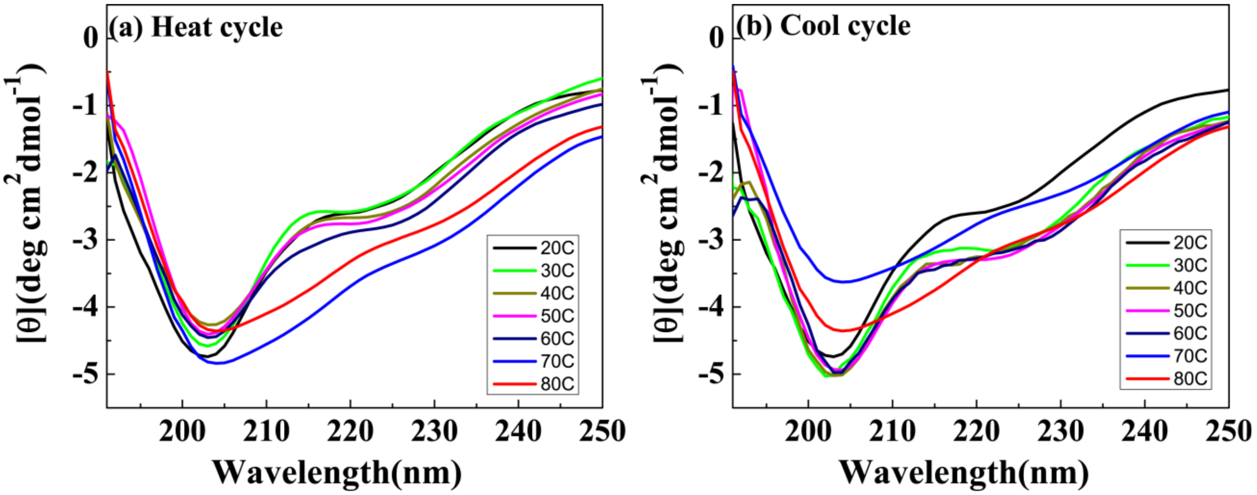

Figure S10. Temperature-dependent circular dichroism (CD) spectra for $[V_7]_{40}$-48hr system during (a) Heat cycle (b) Cool cycle. During heat cycle the characteristic valleys at ~ 203 and 225 nm corresponding to β-sheets persist from 20°C to 60°C. At 70°C and 80°C both the valleys almost vanishes indicative of disaapearnce of β-sheets in $[V_7]_{40}$-48hr system. During cool cycle the valleys corresponding to β-sheets reappeared at 60°C and persists upto 20°C which indicates reversible behavior of β-sheets during heat and cool cycle.

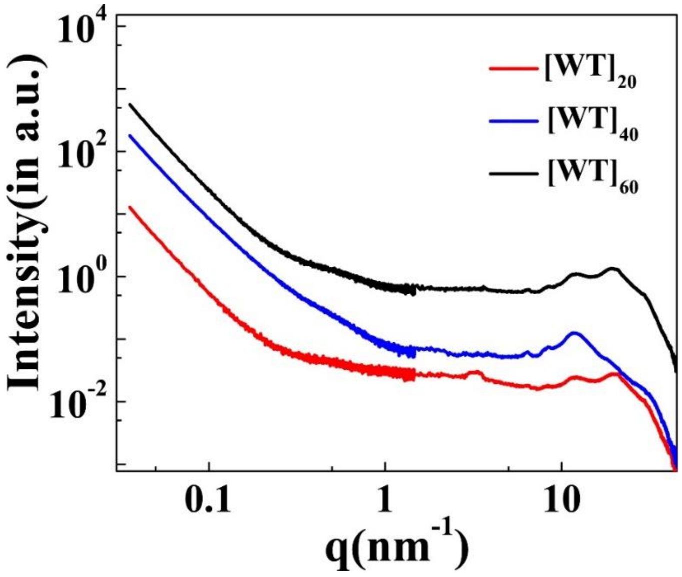

Figure S11. SAXS/WAXS profiles of $(GRGDSPYS)_n$ A-IDPs, after t_{80} = 24 hr, having different numbers of octapeptide repeats, n = 20, 40, 60 for $[WT]_{20}$, $[WT]_{40,}$ and $[WT]_{60}$ respectively.

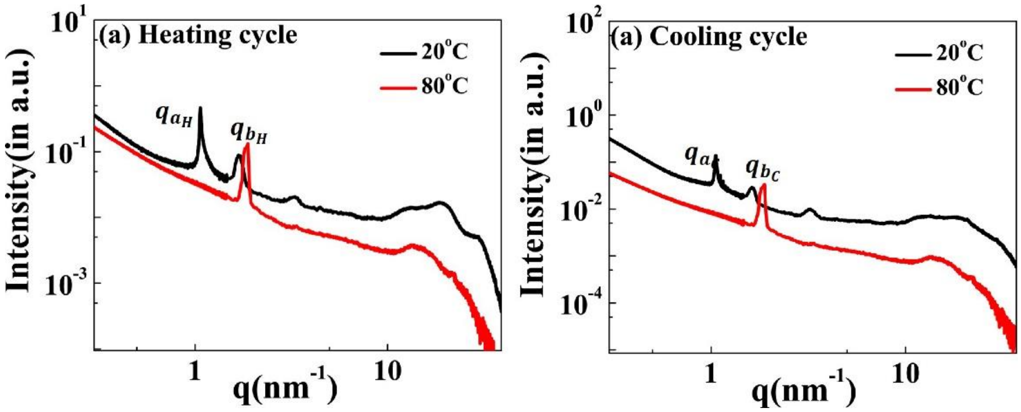

Figure S12. Scattering profiles of the $[WT]_{80}$-24hr system at 20°C and 80°C (a) heating and (b) cooling cycle. No higher-order correlation peaks corresponding to q_a and q_b are observed in the heating-cooling cycle, indicating the absence of short-range nanoscopic order.

Material and Methods

Sample coacervation for X-ray scattering

After incubation, we cooled the RLP solutions to 25°C, which resulted in a turbid solution due to the phase separation —coacervation—of the RLPs. A 20 μL aliquot of the RLP solution was added to a 1.8 mm polycarbonate capillary and centrifuged at 1660 RCF in a swinging bucket centrifuge for 10 min to form a thick pellet at the bottom of the capillary. We repeated this step four times (with 20 μL each of the RLP) to obtain a pellet in the polycarbonate capillary from 80 μL of the RLP solution. The capillaries were then flame-sealed and held vertically in a multistage brass block temperature-controlled sample holder that accommodates up to 40 polycarbonate capillaries of 1.8 mm diameter. To understand the difference between samples prepared from the condensate solution or powders, we also acquired the SAXS profiles of lyophilized powder samples of $[WT]_{40}$, $[WT]_{80}$, and $[V_7]_{40}$ RLPS.

X-ray scattering

We performed simultaneous SAXS and WAXS measurements on the RLPs using the I22 beamline of Diamond Light Source, Oxford, United Kingdom, in transmission mode and X-rays at 10 KeV (λ = 1.2398 Angstrom). The SAXS and WAXS patterns, with a sample-detector distance of 9.7 m and 164 mm, respectively, provide the total overlap range of the scattering vector (q); $0.0014 \leq q \leq 4$ Å^{-1} (where $q = 4\pi sin\theta/\lambda$, where 2θ is the scattering angle, and λ is the wavelength). We acquired the X-ray patterns using two 2D detectors connected in series, Pilatus P3-2M for SAXS and Pilatus P3-2M-DLS-L for WAXS (DECTRIS). For powder samples of, $[V_7]_{40}$-24 h and $[WT]_{80}$-48h measurements were performed using an in-house X-ray scattering system, with a Genix3D (Xenocs) low divergence Cu Kα radiation source

(wavelength of $\lambda = 1.54$ Å) with a EIGER R 1M (DECTRIS) detector.[4] Samples were measured inside 1.5 mm polycarbonate capillaries. All 2D measurements were radially integrated using in-house Matlab code (SAXSi) to get the 1D intensity (I)- scattering vector (q) data sets. We carried out three sets of experiments for each RLP under a particular incubation time and results are reproducible.

To understand the UCST phase behavior of these systems, we acquired X-ray data under temperature ramp-up and ramp-down conditions from 20°C to 80°C with a ramp rate of 1°C min^{-1} at every 5°C. The samples were allowed to equilibrate for 15 min at each temperature before data acquisition. To control for background scattering, we also collected the X-ray patterns of the buffer of each sample at the same temperature as for the relevant sample.

Circular Dichroism

We collected the circular dichroism (CD) spectra for solutions of the RLPs using a Chirascan V100 spectrometer from Applied Photophysics (Leatherhead, United Kingdom), equipped with a temperature controller. We acquired the CD spectra for the RLP solutions with different incubation time (detailed above) over a range of 190−250 nm wavelength range using a 0.1 mm pathlength quartz cuvette and smoothed the data using a 15-point Savitzky−Golay filter. The temperature of the sample holder was stabilized at 25°C for 10 min before the CD measurements.

High-resolution scanning electron microscopy (HRSEM)

About 30 µL of each dialyized RLP solution (prepared and incubated similarly as detailed above) were placed on glass coverslips and left to dry under ambient conditions. The samples were fixed in 2.5% glutaraldehyde overnight at 4°C. After glutaraldehyde treatment, the samples were washed three times in PBS and fixed in 1% OsO_4 in the buffer for 1h, followed by a dehydration series with ethanol. Samples were then incubated in absolute ethanol for 30 min, followed by critical point drying and coating with gold. We recorded the micrographs using HRSEM (Zeiss, Gemini SEM 3000).

Matrix-assisted laser desorption/ionization time of flight mass spectrometry (MALDI-TOF-MS)

Samples were analyzed using Bruker Autoflex LRF matrix-assisted laser desorption ionization tandem time of flight mass spectrometer (MALDI-TOF MS). 1 µL of each protein sample (100uM protein in 150mM phosphate buffer, incubated for t = 24h or t = 48h at 80°C) was mixed with 3 µL of a saturated solution of Sinapic acid (SA) matrix and deposited on a ground steel MALDI plate.

Alphafold2

The ab-initio predicted three-dimensional structures of WT and V7 were generated using the AlphaFold Colab server that predicts protein structures starting from their sequences using a slightly simplified version of AlphaFold v2.0.[2] This server does not consider existing structural templates.

Fourier-transform Infrared (FTIR) spectroscopy

A sample of 20μl was deposited on 19mm KBr cards (Sigma-Aldrich), then allowed to air dry. FTIR spectra were collected using nitrogen-purged Nicolet Nexus 470 FTIR spectrometer equipped with a deutrated triglycine sulphate detector. Measurements were performed using a 4cm^{-1} resolution and by averaging 64 scans.

1. Micsonai, A. *et al.* BeStSel: a web server for accurate protein secondary structure prediction and fold recognition from the circular dichroism spectra. *Nucleic Acids Res.* **46**, W315–W322 (2018).

2. Jumper, J. *et al.* Highly accurate protein structure prediction with AlphaFold. *Nat. 2021 5967873* **596**, 583–589 (2021).

3. Masunaga, H., Sakurai, K., Akiba, I., Ito, K. & Takata, M. Accurate measurements of intrinsic scattering from window materials by use of a vacuum camera. *J. Appl. Cryst.* **46**, 577–579 (2013).

4. Li, Y., Beck, R., Huang, T., Choi, M. C. & Divinagracia, M. Scatterless hybrid metal-single-crystal slit for small-angle X-ray scattering and high-resolution X-ray diffraction. *J. Appl. Crystallogr.* **41**, 1134–1139 (2008).
